# MicroRNA Expression Signature in Human Calcific Aortic Valve Disease

**DOI:** 10.1155/2017/4820275

**Published:** 2017-04-11

**Authors:** Hui Wang, Jing Shi, Beibei Li, Qiulian Zhou, Xiangqing Kong, Yihua Bei

**Affiliations:** ^1^Department of Cardiology, The First Affiliated Hospital of Nanjing Medical University, Nanjing 210029, China; ^2^Cardiac Regeneration and Ageing Lab, School of Life Science, Shanghai University, Shanghai 200444, China

## Abstract

Altered microRNA (miRNA, miR) expression has been related to many disease processes; however, the miRNA expression signature in calcific aortic valve disease (CAVD) is unclear. In this study, microarrays were used to determine the miRNA expression signature of tissue samples from healthy individuals (*n* = 4) and patients with CAVD (*n* = 4). TargetScan, PITA, and microRNAorg 3-way databases were used to predict the potential target genes. DIANA-miRPath was used to incorporate the aberrant miRNAs into gene pathways. miRNA microarrays identified 92 differentially expressed miRNAs in CAVD tissues. The principal component analysis (PCA) of these samples and the unsupervised hierarchical clustering analysis based on the 92 aberrantly expressed miRNAs noted that miRNA expression could be categorized into two well-defined clusters that corresponded to healthy control and CAVD. Bioinformatic analysis showed the miRNA targets and potential molecular pathways. Collectively, our study reported the miRNA expression signature in CAVD and may provide potential therapeutic targets for CAVD.

## 1. Background

Valve diseases continue to occur in many patients with significant morbidity and mortality. The age-adjusted prevalence of moderate or severe valve diseases was estimated at 2.5% [1]. Calcific aortic valve disease (CAVD) is the most common valve heart disease in the elderly and a leading cause of aortic stenosis [2]. In developing countries, CAVD represents a major cause for surgical valve replacement [3]. As a result of rising life expectancy and ageing populations, the burden of CAVD will significantly increase in the near future.

While CAVD was originally thought to be a degenerative process with passive deposition of calcium phosphate in the valve occurring with age, it now appears to be a complex and actively regulated progress mediated by inflammation, cell apoptosis, lipid deposition, renin-angiotensin system activation, extracellular matrix remodeling, and bone formation [4–6]. To better monitor progression of CAVD and identify the most appropriate instances for surgical intervention, biomarkers can be serially monitored. Such biomarkers would represent objective laboratory measurements, as older patients with CAVD might have atypical symptoms associated with comorbidities such as pulmonary disease or orthopaedic disabilities [7, 8].

MicroRNAs (miRNAs, miRs) are endogenous, small, single-stranded, 21–25 nucleotide noncoding RNAs, regulating target gene expressions by hybridizing to messenger RNAs (mRNAs). An individual miRNA is able to target tens to hundreds of genes while a single gene can also be targeted by lots of miRNAs [9]. Since miRNAs play critical roles in many physiological processes, increasing reports indicate that a distinct pattern of altered miRNA expressions may be linked to specific disease processes [10–15]. We previously reported the miRNA expression signature in degenerative aortic stenosis [14]. In this study, we explored the miRNA expression signature in CAVD.

## 2. Materials and Methods

### 2.1. Tissue Sample Collection and RNA Isolation

This study was officially approved by the Ethics Committees of the First Affiliated Hospital of Nanjing Medical University and conformed to the principles outlined in the Declaration of Helsinki. All written informed consent was obtained from patients, and parents where applicable. Tissue samples from four healthy subjects were collected from prospective multiorgan donors in cases because of technical reasons that prevented transplantation, while stenotic aortic valve samples were obtained from four patients who underwent surgical valve replacement for aortic stenosis. All samples were examined by gross examination, and microscopic examination of hematoxylin and eosin-stained cryosections was conducted to confirm the presence/absence of CAVD. Tissue samples harvested from subject donors were snap-frozen in liquid nitrogen, and RNA was then isolated using the RNeasy Mini Kit (Qiagen, Hilden, Germany) according to the manufacturer's instructions.

### 2.2. miRNA Microarray Analysis

Total RNAs were isolated from heart tissues using mirVana™ RNA Isolation Kit, quantified by NanoDrop ND-2100 (Thermo Scientific), and controlled for RNA integrity using Agilent Bioanalyzer 2100 (Agilent Technologies) according to the manufacturer's instructions. miRNA profiling was performed with OE Biotech's (Shanghai, China) miRNA microarray service. The arrays from the control group are the same as we previously used [14].

### 2.3. Bioinformatic Analysis

TargetScan, PITA, and microRNAorg 3-way databases were used to identify potential human miRNA target genes and a Venn diagram was made to provide relations among the 3 databases. DIANA tool miRPath v2.0, a web-based analysis tool, was used for pathway enrichment analysis for the miRNA set identified [16]. DIANA tool miRPath assigns Kyoto Encyclopedia of Genes and Genomes (KEGG) pathway with the significance level determined by the number of target genes affected by the identified microRNAs.

### 2.4. Statistical Analysis

Independent Student's *t*-test was used to determine whether there were any significant differences between the miRNA expression profiles between two groups. *P* values less than 0.05 (*P* < 0.05) were considered to be statistically significant. Significant data were further analyzed by clustering, and the expression profiles were visualized with GeneSpring 10.0 (Agilent Technology).

## 3. Results

### 3.1. Principal Component Analysis of miRNA Expression Profiles

**Figure 1:**
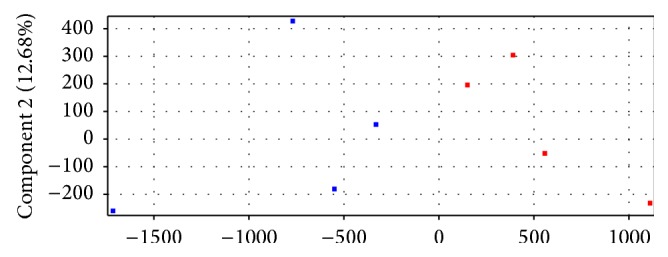
PCA of the miRNA profiles in CAVD tissue samples and control subjects. Red dots represent samples from control group, while blue dots represent samples from CAVD group.

Principal component analysis (PCA) is a mathematical algorithm that reduces the dimensionality of the data while retaining most of the variation in the data set [17]. Dots in two colors separated in two axes based on the differences of the data, suggesting that samples in this study were prepared appropriately and could be grouped as CAVD or healthy control (Figure 1).

### 3.2. Unsupervised Hierarchical Cluster Analysis of miRNA Microarray Data

**Figure 2:**
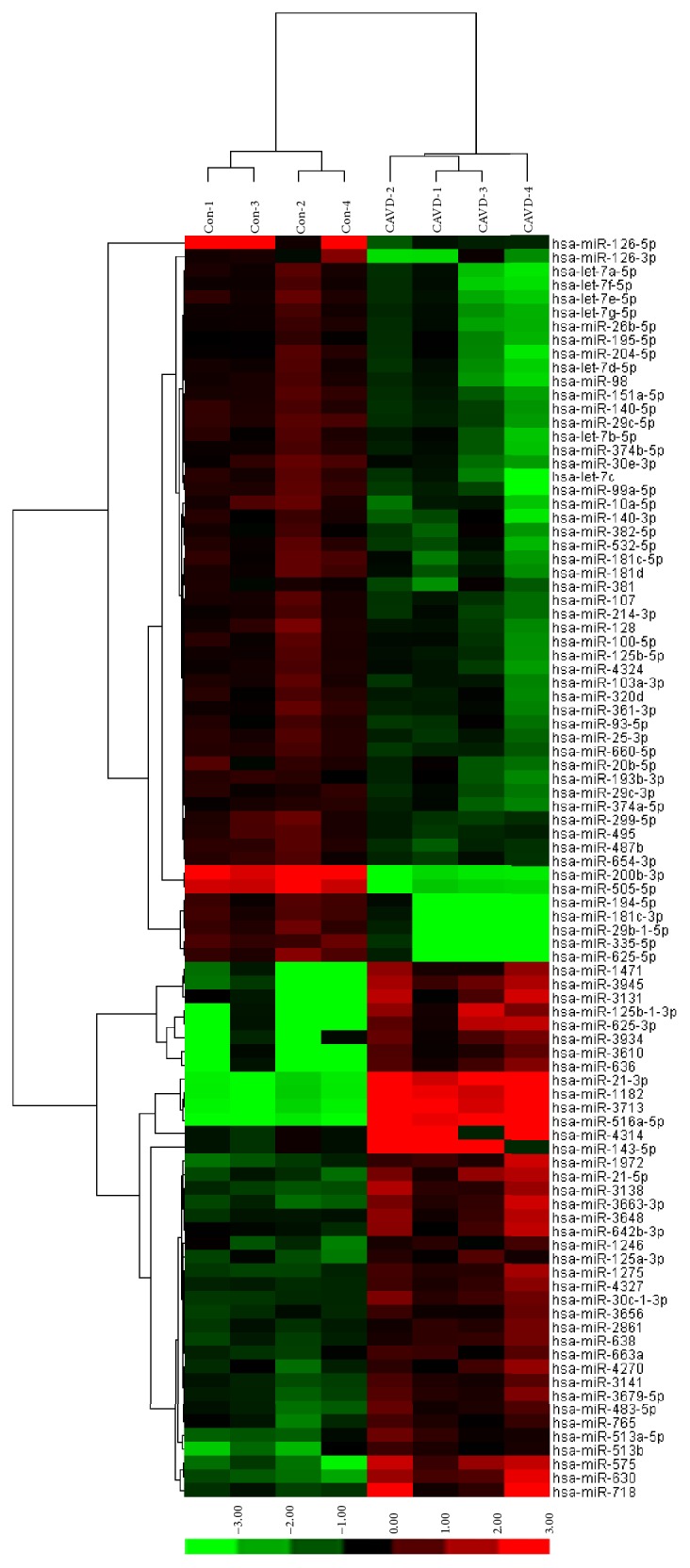
Unsupervised hierarchical clustering identified two distinct groups (CAVD versus control) based on their miRNA expression profile. Sample names are listed at top. The names of the significantly altered (*P* value < 0.05) miRNAs are shown at right. Ninety-two miRNAs were expressed differently between the two groups.

miRNA arrays identified 92 miRNAs with a statistically significant differential expression of 2.0-fold or greater in CAVD samples relative to normal controls. Fifty-three miRNAs were underexpressed and 39 were overexpressed in aortic tissue from CAVD patients (Table 1). Unsupervised hierarchic clustering of the two groups was performed on the 92 differently expressed miRNAs and displayed as heatmap (Figure 2).

### 3.3. Target Genes Analysis

**Figure 3:**
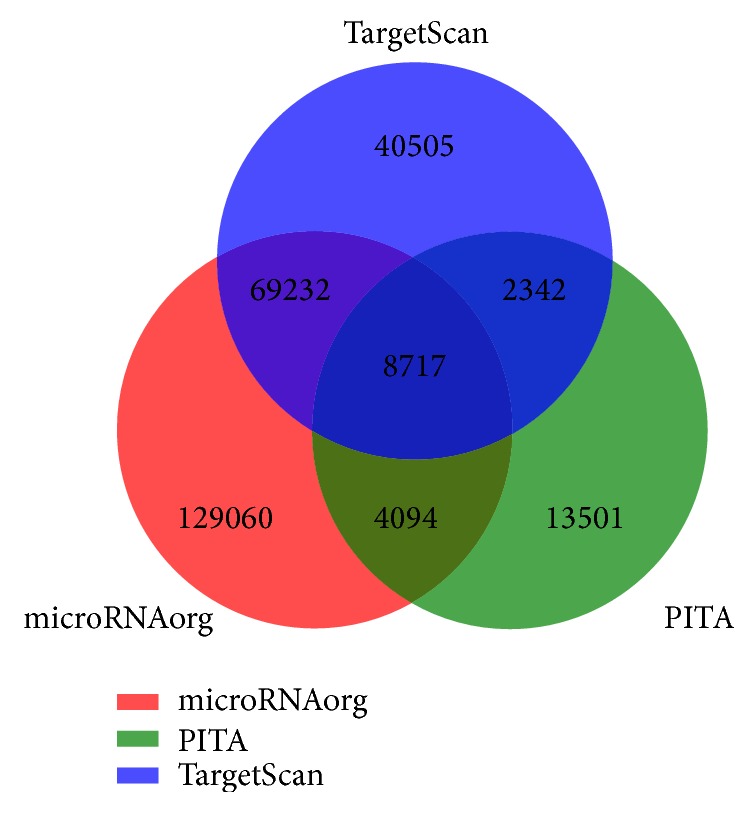
The red, green, and blue sets stand for target genes predicted by database microRNAorg, TargetScan, and PITA, respectively.

MicroRNAorg, TargetScan, and PITA were used to predict the targets of differentially expressed miRNAs in CAVD samples. A Venn diagram was made to highlight the relations among the three databases. There are 8717 genes overlapping by all three sets, which are most likely to be targets of miRNAs in patients with CAVD (Figure 3).

### 3.4. DIANA miRNA Pathway Analysis

To better understand the putative mechanisms underlying CAVD, we used DIANA-miRPath (v2.0), a web-based server developed to identify the potential cellular pathways regulated by microRNAs. We first evaluated downregulated miRNAs in CAVD samples compared to control samples. The potential affected pathways included the following: cell cycle, PI3K-Akt signaling pathway, ECM-receptor interaction, HIF-1 signaling pathway, p53 signaling pathway, ErbB signaling pathway, Neurotrophin signaling pathway, focal adhesion, and DNA replication (Table 2). Upregulated miRNAs were also used to generate the potential affected pathways by DIANA-miRPath and identified p53 signaling pathway, HIF-1 signaling pathway, valine, leucine, and isoleucine biosynthesis, ErbB signaling pathway, cell cycle, mTOR signaling pathway, MAPK signaling pathway, PI3K-Akt signaling pathway, Wnt signaling pathway, synthesis and degradation of ketone bodies, TGF-beta signaling pathway, basal transcription factors, glycerophospholipid metabolism, hypertrophic cardiomyopathy (HCM), focal adhesion, circadian rhythm, mismatch repair, lysine degradation, and butanoate metabolism (Table 3).

## 4. Discussion

miRNAs have been shown to be critical regulators in cardiovascular diseases [18–25]. However, there are no reports revealing distinct miRNA expression signatures in the CAVD patients and healthy controls. In this study, we identified global changes in the miRNA expression profile in CAVD and healthy control. Calcific aortic valve stenosis is characterized by lipid accumulation, inflammation, formation of plaque neovessels, hemorrhages, neointimal formation, vascular fibrosis, and ectopic calcification [4, 26]. Previous studies have shown that miRNAs play crucial roles in those processes such as angiogenesis, fibrogenesis, proliferation, and apoptosis [9].

miR-126 is one of the most abundantly expressed microRNAs in endothelial cells (ECs) [27]. Upregulation of miR-126 increases EC survival, decreases EC apoptosis, and prevents reactive oxygen species (ROS) mediated endothelial damage [28]. Our findings of decreased miR-126 in CAVD may suggest a detrimental effect in human calcific aortic valve.

The differentially expressed miRNAs identified in the current study also included many profibrotic miRNAs such as miR-21 and miR-125b that might contribute to CAVD by promoting fibrosis. Several expression profiling studies identify that increased level of miR-21-5p in cardiac fibroblasts promotes cardiac fibrosis via its target genes: phosphatase and tensin homolog (PTEN) [29] and Sprouty-1 (*Spry*1) [30]. Additionally, miR-125b is a novel regulator of cardiac fibrogenesis, proliferation, and fibroblast-to-myofibroblast transition. Nagpal et al. demonstrated the upregulation of miR-125b in fibrotic human heart and murine models of cardiac fibrosis [31].

Interestingly, our miRNA array data revealed that several members of the let-7 (let-7a, let-7b, let-7c, let-7d, let-7e, let-7f, and let-7g) were downregulated in calcific aortic valve. Let-7g targets the genes related to vascular smooth muscle cell (VSMC) functions, including ROS, autophagy-related proteins (expression of beclin-1, LC3-II, and Atg5), and apoptosis-related proteins (expression of caspase-3, Bax, Bcl-2, and Bcl-xL) [32]. Let-7 family members might directly influence aortic valve sclerosis by regulating the proliferation, migration, autophagy, and apoptosis of VSMC, which have been implicated in the progression of CAVD [4, 26].

The abnormal expression of miR-21-5p was found in many cardiovascular diseases [33]. Programmed cell death 4 (PDCD4) is identified as a direct target gene of miR-21-5p. It has been reported that miR-21-5p prevented cardiomyocyte apoptosis in ischaemia/reperfusion heart model through PDCD4 repression [34]. Furthermore, miR-21/PDCD4 pathway was proved to be involved in cardiac valvulogenesis by regulating endothelial cell migration [35]. In our work, miR-21-5p was upregulated in calcific aortic valve which indicates that miR-21-5p might take part in CAVD. However, the effects and mechanisms of miR-21-5p on calcific aortic valve are still to be investigated in further studies.

A comprehensive knowledge of miRNA expression is essential to improve our understanding of this disease. This study provides the first evidence that there exists a distinct miRNA expression signature in individuals with CAVD, as compared to healthy controls. There are 92 differently expressed miRNAs in the CAVD patients compared with healthy controls by miRNA arrays. PCA and unsupervised hierarchical clustering with these miRNAs demonstrates that this profile could accurately classify the samples according to their disease status. Moreover, bioinformatic tools indicate that the differential expression of miRNAs could be linked to several targets and pathways.

As a limitation of our study, the exact pathways by which dysregulated miRNAs cause CAVD in human remain elusive. Further studies are required to fully characterize the function of candidate miRNAs.

## 5. Conclusions

Taken together, the current study provides insight into the importance of microRNA expression signature in CAVD. A deeper understanding of the molecular alternations in CAVD may provide potential targets for future clinical applications.

## Figures and Tables

**Table 1:** Fifty-three underexpressed and 39 overexpressed miRNAs in aortic tissue from CAVD patients compared to the control group.

Systematic name	*P* value	Fold change	Regulation
hsa-miR-3656	0.011202	2.0143232	Up
hsa-miR-765	0.033639	2.0401733	Up
hsa-miR-2861	0.008741	2.0782373	Up
hsa-miR-663a	0.00633	2.0819619	Up
hsa-miR-1246	0.027045	2.1549542	Up
hsa-miR-3141	0.002969	2.2772849	Up
hsa-miR-125a-3p	0.012002	2.3428166	Up
hsa-miR-4327	0.004807	2.367507	Up
hsa-miR-638	0.002785	2.3686504	Up
hsa-miR-4270	0.030574	2.4421182	Up
hsa-miR-642b-3p	0.034575	2.5763547	Up
hsa-miR-513a-5p	0.007538	2.6546612	Up
hsa-miR-483-5p	0.008099	2.655595	Up
hsa-miR-3679-5p	0.004016	2.7413363	Up
hsa-miR-3648	0.017053	2.8097122	Up
hsa-miR-30c-1-3p	7.64E-04	2.893416	Up
hsa-miR-1275	0.005457	2.9556682	Up
hsa-miR-513b	0.020604	3.4257321	Up
hsa-miR-1972	0.011389	3.689852	Up
hsa-miR-3138	0.004076	3.974949	Up
hsa-miR-3663-3p	0.005874	4.314916	Up
hsa-miR-21-5p	0.003826	4.317176	Up
hsa-miR-718	0.031718	4.957687	Up
hsa-miR-630	0.001304	7.3841376	Up
hsa-miR-575	0.001586	10.079804	Up
hsa-miR-3934	0.048827	13.458452	Up
hsa-miR-143-5p	0.033632	16.2909	Up
hsa-miR-3131	0.047501	22.065104	Up
hsa-miR-125b-1-3p	0.00519	23.412596	Up
hsa-miR-625-3p	0.005867	23.648891	Up
hsa-miR-1471	0.035145	25.31259	Up
hsa-miR-4314	0.032125	27.92129	Up
hsa-miR-636	0.00923	28.36478	Up
hsa-miR-3945	0.018336	38.864952	Up
hsa-miR-3610	0.014352	49.196358	Up
hsa-miR-1182	1.46E-05	73.30232	Up
hsa-miR-3713	2.44E-05	117.90961	Up
hsa-miR-21-3p	5.95E-05	149.08258	Up
hsa-miR-516a-5p	2.88E-05	155.79349	Up
hsa-miR-654-3p	0.002559	2.0230756	Down
hsa-miR-93-5p	0.020279	2.0238087	Down
hsa-miR-320d	0.038247	2.052202	Down
hsa-miR-381	0.035566	2.0584567	Down
hsa-miR-214-3p	0.011756	2.0957778	Down
hsa-miR-125b-5p	0.036771	2.1063256	Down
hsa-miR-361-3p	0.036059	2.1256602	Down
hsa-miR-29c-3p	0.009743	2.1358023	Down
hsa-miR-495	0.001289	2.1425073	Down
hsa-miR-374a-5p	0.01904	2.1439137	Down
hsa-miR-20b-5p	0.027368	2.1694279	Down
hsa-miR-382-5p	0.043289	2.1756916	Down
hsa-miR-4324	0.039752	2.178068	Down
hsa-miR-25-3p	0.004579	2.1911306	Down
hsa-miR-100-5p	0.035509	2.1913323	Down
hsa-miR-193b-3p	0.022676	2.191656	Down
hsa-miR-107	0.007782	2.2016506	Down
hsa-miR-660-5p	8.68E-04	2.2094207	Down
hsa-miR-103a-3p	0.020178	2.2370007	Down
hsa-miR-195-5p	0.049708	2.3292358	Down
hsa-miR-299-5p	0.002066	2.3574395	Down
hsa-miR-487b	7.71E-04	2.422462	Down
hsa-miR-128	0.022531	2.4691415	Down
hsa-miR-181d	0.019566	2.5412066	Down
hsa-miR-374b-5p	0.038373	2.5474217	Down
hsa-let-7b-5p	0.042981	2.6197023	Down
hsa-miR-140-5p	0.005972	2.639281	Down
hsa-let-7g-5p	0.02818	2.7145965	Down
hsa-miR-151a-5p	0.010444	2.7681546	Down
hsa-miR-532-5p	0.020008	2.7994845	Down
hsa-miR-26b-5p	0.025414	2.8088717	Down
hsa-miR-30e-3p	0.020305	2.8739653	Down
hsa-miR-140-3p	0.040144	2.8943768	Down
hsa-miR-29c-5p	0.004665	2.9975233	Down
hsa-miR-181c-5p	0.015322	3.0353231	Down
hsa-miR-204-5p	0.044941	3.0471704	Down
hsa-let-7d-5p	0.025658	3.0987353	Down
hsa-miR-98	0.027495	3.2012997	Down
hsa-miR-10a-5p	0.019225	3.3482268	Down
hsa-let-7f-5p	0.037282	3.4332418	Down
hsa-let-7e-5p	0.018337	3.6489105	Down
hsa-let-7a-5p	0.029403	3.6912477	Down
hsa-miR-99a-5p	0.035923	3.8172672	Down
hsa-let-7c	0.032672	3.9863558	Down
hsa-miR-126-3p	0.026073	4.528461	Down
hsa-miR-29b-1-5p	0.006566	15.589992	Down
hsa-miR-181c-3p	0.008219	16.225191	Down
hsa-miR-194-5p	0.011511	20.940771	Down
hsa-miR-335-5p	0.006273	36.750603	Down
hsa-miR-126-5p	0.015298	38.692142	Down
hsa-miR-505-5p	1.18E-05	42.592148	Down
hsa-miR-625-5p	0.008835	43.434204	Down
hsa-miR-200b-3p	1.60E-06	70.24472	Down

**Table 2:** The pathways incorporated by downregulated microRNAs in CAVD.

KEGG pathway	*P* value	Genes	miRNAs
Cell cycle	<1*E* − 16	48	19
PI3K-Akt signaling pathway	2.22*E* − 16	95	17
ECM-receptor interaction	1.85*E* − 12	9	4
HIF-1 signaling pathway	8.63*E* − 10	30	15
p53 signaling pathway	1.49*E* − 08	28	14
ErbB signaling pathway	7.14*E* − 05	23	12
Neurotrophin signaling pathway	0.00249	19	10
Focal adhesion	0.0029	19	6
DNA replication	0.013417	19	2

**Table 3:** The pathways affected by upregulation of specific microRNAs in CAVD.

KEGG pathway	*P* value	Genes	miRNAs
p53 signaling pathway	5.63*E* − 09	11	2
HIF-1 signaling pathway	0.00023	10	2
Valine, leucine, and isoleucine biosynthesis	0.00227	1	1
ErbB signaling pathway	0.003959	5	1
Cell cycle	0.003959	10	2
mTOR signaling pathway	0.004909	6	1
MAPK signaling pathway	0.005193	15	2
PI3K-Akt signaling pathway	0.005193	17	2
Wnt signaling pathway	0.008586	10	1
Synthesis and degradation of ketone bodies	0.009366	2	1
TGF-beta signaling pathway	0.021216	7	1
Basal transcription factors	0.029417	4	1
Glycerophospholipid metabolism	0.030271	8	1
Hypertrophic cardiomyopathy (HCM)	0.030271	6	1
Focal adhesion	0.030271	11	2
Circadian rhythm	0.030848	3	1
Mismatch repair	0.034518	2	1
Lysine degradation	0.038471	4	1
Butanoate metabolism	0.038615	3	1

Specific types of cancers and infections were not included.
